# Emergence or improved detection of Japanese encephalitis virus in the Himalayan highlands?

**DOI:** 10.1093/trstmh/trw012

**Published:** 2016-03-07

**Authors:** Matthew Baylis, Christopher M. Barker, Cyril Caminade, Bhoj R. Joshi, Ganesh R. Pant, Ajit Rayamajhi, William K. Reisen, Daniel E. Impoinvil

**Affiliations:** a Institute of Infection and global Health, University of Liverpool, Liverpool, UK; b Health Protection Research Unit in Emerging and Zoonotic Infections, University of Liverpool, UK; c Davis Arbovirus Research and Training, Department of Pathology, Microbiology, and Immunology, School of Veterinary Medicine, University of California, Davis, CA 95616, USA; d Centre for Environmental and Agricultural Policy Research, Extension and Development (CEAPRED), Nayabato, Lalitpur, Nepal; e Department of Animal Science, Veterinary Science and Fisheries, Agriculture and Forestry University, Chitwan, Nepal; f Department of Pediatrics, National Academy of Medical Sciences, Kanti Children's Hospital, Maharajgunj, Kathmandu, Nepal

**Keywords:** Climate change, Emergence, Japanese encephalitis, JEV, Mosquito, Nepal

## Abstract

The emergence of Japanese encephalitis virus (JEV) in the Himalayan highlands is of significant veterinary and public health concern and may be related to climate warming and anthropogenic landscape change, or simply improved surveillance. To investigate this phenomenon, a One Health approach focusing on the phylogeography of JEV, the distribution and abundance of the mosquito vectors, and seroprevalence in humans and animal reservoirs would be useful to understand the epidemiology of Japanese encephalitis in highland areas.

Japanese encephalitis (JE) is a zoonotic neuro-tropic vectorborne disease that remains a major cause of viral encephalitis in Asia.^1^ The distribution of Japanese encephalitis virus (JEV) transmission risk extends latitudinally from eastern Russia to the northern tip of Queensland, Australia, and longitudinally from eastern Pakistan to Papua New Guinea. An important reason for JEV's persistence, despite the availability of an effective human vaccine since 1954,^1^ is that the causative virus is sustained in an enzootic cycle and non-vaccinated humans (especially children) are at continual risk from infectious mosquito bites. Maintenance of JEV transmission purportedly involves ardeids (herons, egrets) and rice-paddy mosquitoes, with epizootic transmission among pigs that amplify the virus and serve as a source of infection for peridomestic mosquitoes that infect humans. The mosquito *Culex tritaeniorhynchus* is considered to be the primary vector of JEV due to its abundance throughout the JEV geographical range, catholic feeding habit, which includes birds, pigs and humans, high frequency of infection detected by entomological surveys, and high competency for virus transmission in experimental infection studies.^1^ Other mosquito species may be locally important.^2^ Infection in sows frequently results in abortion, whereas humans and horses are dead-end hosts, not contributing to transmission, but may suffer severe neurological disease.^1^ The spill-over of virus from the pig epizootic cycle to humans and horses indicates the need for a One Health approach to integrate medical and veterinary measures for control.

Complicating measures to focus JE control are recent reports of its spread to higher elevations in the Himalayas. In Nepal and Tibet, China, studies suggest the recent emergence of JEV at elevations from 1000–3000 meters a.s.l.^2–4^ These studies provide evidence of recent infection in pigs and humans, with no reported history of movement from low elevation JE-endemic regions. *C. tritaeniorhynchus* is well-established at lower elevations (<2000 m a.s.l), but has not been found at higher elevations (>2000 m a.s.l),^2^ despite its ability to survive cold winters in diapause.^1^ Although these studies document the presence of JEV at higher elevations, and the possible involvement of other vector species,^2^ it is not clear if this represents the emergence of novel transmission at high elevation due to climate warming or the discovery of pre-existing seasonal transmission through extended surveillance.

Climate change in the Himalayas or, possibly, the effects of short-term climate variability are plausible explanations for the spread of JEV to high elevations. Warming trends would allow the upslope expansion of suitable climatic conditions for the vector and virus replication within it; and also rice production, providing breeding sites for the vector and feeding grounds for ardeids; and pig production, providing amplifying hosts for JEV.^5, 6^ Temperature increases are implicated as drivers of the spread of other vector-borne diseases to areas of higher elevation, such as malaria in highland areas of South America and Africa.

Alternatively, JEV may have been present in highland regions for some time and is simply being detected because of improved disease surveillance, diagnostics and health systems. Without laboratory confirmation, JE is often grouped within acute encephalitis syndrome (AES) cases, where the disease aetiology of the patient is not specified. With better health services, JE may be detected more frequently, despite no actual change in disease incidence. For instance, reports of JE in the highlands of Nepal coincided with the introduction of AES surveillance in 2004 through a national sentinel surveillance network, conducted by the government of Nepal, and supported by the WHO.^7^ Similarly, the expansion of China's public health and research infrastructure corresponded to discoveries of JE in Tibet. Vaccination programs may also confound the perceived distribution of transmission. For example, the vaccination strategy of Nepal provided universal vaccination in high-risk districts in the lowland (‘Terai’) regions, but only targeted vaccination in moderate risk, hill regions, and no vaccination in the lowest risk, high elevation, mountain regions. The shift in spatial clustering of Nepal's JE cases, from highly endemic districts of Terai to the higher elevation Kathmandu Valley (Figure 1) after the mass vaccination in 2006,^8^ could be attributed to the reduction of cases in high-risk Terai districts through more intense vaccination relative to the other districts,^9^ as well as to the natural temporal cycling of JEV and its vectors.

**Figure 1.**
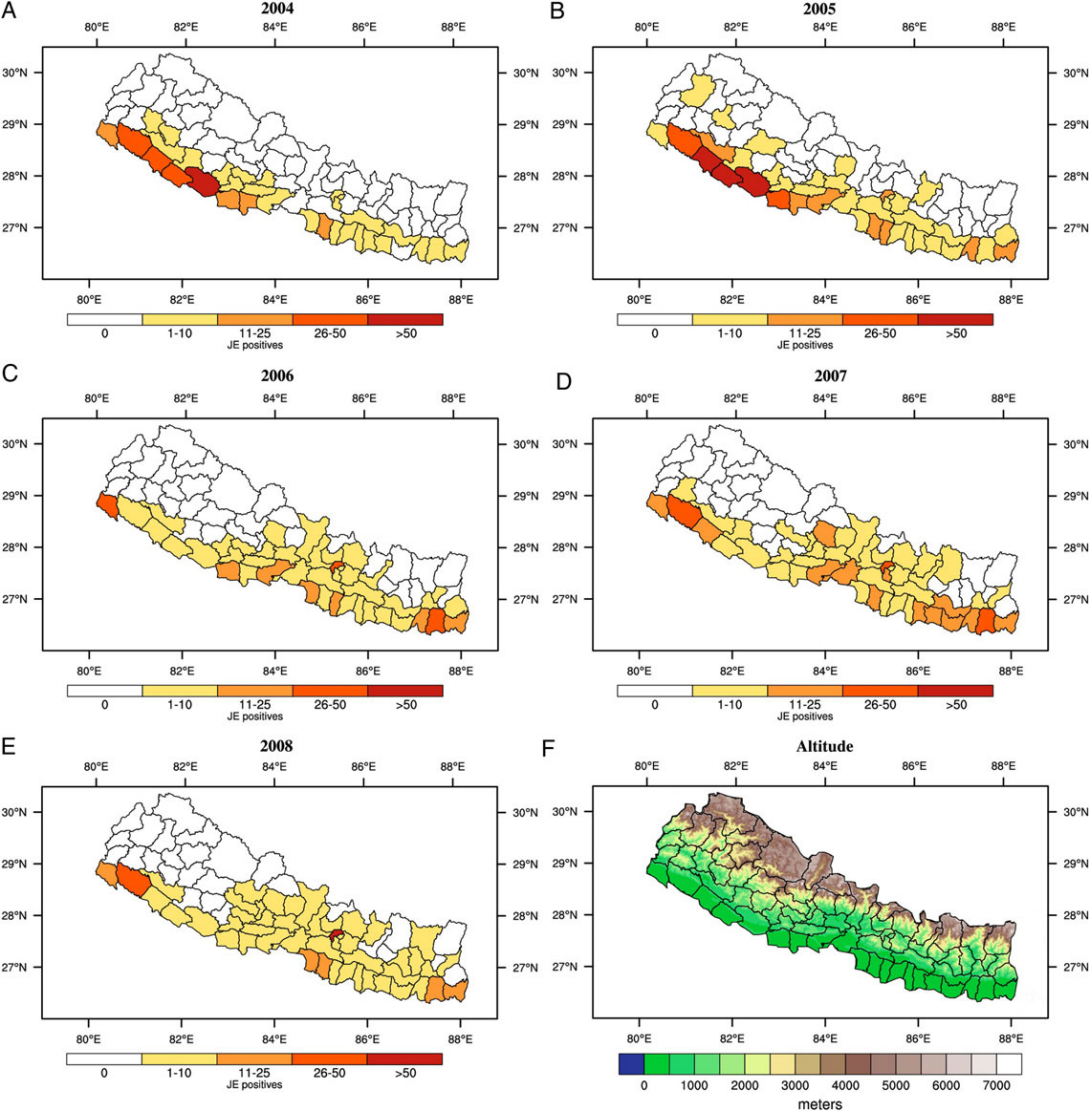
Number of lab confirmed cases of Japanese encephalitis (JE) at the district level for A) 2004, B) 2005, C) 2006, D) 2007 and E) 2008. See^10^ for more information on the clinical data. F) Altitude (metres).

Phylogeographic studies of JEV and its vectors collected from highland and lowland areas may be a useful way to identify recent and past pathogen transmission and spread. Tools such as Bayesian Evolutionary Analysis Sampling of Trees (BEAST) are being used to estimate the time since emergence of JEV,^10^ and could be applied to determine the origin of mosquito populations as well. Such an approach, if applied to JEV and vectors in highlands, may provide information on whether emergence is recent or recently discovered. As JE is zoonotic, investigations of pathogen transmission in animals, particularly birds and pigs, may be helpful in understanding emerging transmission. As birds are a reservoir for JEV,^1^ comparing seroprevalence of birds from the lowland and highland regions may provide insights into the relative extent of transmission in these two regions. Such studies would also help bridge the gap between medical and veterinary control efforts and wildlife health.

In conclusion, several factors must be considered when addressing the apparent recent emergence of JE at high elevation in the Himalayas. It is currently not clear whether increased detection of JE in the highlands is due to change in environmental factors leading to its upslope spread, or is a consequence of extended surveillance. Phylogenetic analytical methods, sero-epidemiological studies, and integrated medical and veterinary surveillance efforts will improve understanding of the distribution of transmission. By developing a clear understanding of transmission, ministries of health can be better informed and prepared to deliver effective public health interventions.
